# Supplementary material for: Modeling glioblastoma heterogeneity as a dynamic network of cell states
Source: Mol Syst Biol. 2021 Sep 16;17(9):e10105. doi: 10.15252/msb.202010105 (PMC8444284; doi:10.15252/msb.202010105)
Supplement: Supplementary file 5 — Source Data for Figure 3 [file MSB-17-e10105-s001.zip › Figure3A_sourcedata/GSEA_3065/nosigdb_state5.GseaPreranked.1622718435914/FELDMAN_2020_S-G2.html]

Details for gene set FELDMAN\_2020\_S-G2[GSEA]

|  || Dataset | state5 |
| Phenotype | NoPhenotypeAvailable |
| Upregulated in class | na\_neg |
| GeneSet | FELDMAN\_2020\_S-G2 |
| Enrichment Score (ES) | -0.5286256 |
| Normalized Enrichment Score (NES) | -2.0355892 |
| Nominal p-value | 0.0 |
| FDR q-value | 0.0 |
| FWER p-Value | 0.0 |
Table: GSEA Results Summary

  

Fig 1: Enrichment plot: FELDMAN\_2020\_S-G2      
 Profile of the Running ES Score & Positions of GeneSet Members on the Rank Ordered List

  

| PROBE | GENE SYMBOL | GENE\_TITLE | RANK IN GENE LIST | RANK METRIC SCORE | RUNNING ES | CORE ENRICHMENT || 1 | MALAT1 |  |  | 0 | 1.196 | 0.0133 | No |
| 2 | PTPRZ1 |  |  | 12 | 0.862 | 0.0217 | No |
| 3 | SCRG1 |  |  | 36 | 0.715 | 0.0270 | No |
| 4 | PLAT |  |  | 37 | 0.714 | 0.0349 | No |
| 5 | GPM6B |  |  | 41 | 0.702 | 0.0424 | No |
| 6 | LAPTM4A |  |  | 51 | 0.641 | 0.0485 | No |
| 7 | MIAT |  |  | 58 | 0.619 | 0.0547 | No |
| 8 | GPM6A |  |  | 60 | 0.609 | 0.0613 | No |
| 9 | PRCP |  |  | 103 | 0.523 | 0.0623 | No |
| 10 | ITM2B |  |  | 117 | 0.509 | 0.0665 | No |
| 11 | TTYH1 |  |  | 141 | 0.489 | 0.0693 | No |
| 12 | TMEM59 |  |  | 151 | 0.481 | 0.0736 | No |
| 13 | CLU |  |  | 162 | 0.474 | 0.0777 | No |
| 14 | SCD5 |  |  | 173 | 0.460 | 0.0817 | No |
| 15 | GGH |  |  | 184 | 0.453 | 0.0856 | No |
| 16 | CTGF |  |  | 199 | 0.440 | 0.0888 | No |
| 17 | PSAP |  |  | 227 | 0.420 | 0.0904 | No |
| 18 | ARL6IP5 |  |  | 256 | 0.406 | 0.0917 | No |
| 19 | CDH2 |  |  | 261 | 0.403 | 0.0957 | No |
| 20 | HELLS |  |  | 280 | 0.393 | 0.0980 | No |
| 21 | SPARC |  |  | 327 | 0.370 | 0.0968 | No |
| 22 | CNPY2 |  |  | 336 | 0.365 | 0.1000 | No |
| 23 | TM7SF2 |  |  | 352 | 0.360 | 0.1022 | No |
| 24 | RABAC1 |  |  | 358 | 0.358 | 0.1057 | No |
| 25 | NDFIP1 |  |  | 386 | 0.348 | 0.1064 | No |
| 26 | TNFRSF12A |  |  | 615 | 0.282 | 0.0832 | No |
| 27 | ORC6 |  |  | 806 | 0.240 | 0.0639 | No |
| 28 | DCBLD2 |  |  | 863 | 0.230 | 0.0600 | No |
| 29 | HES4 |  |  | 977 | 0.213 | 0.0494 | No |
| 30 | ATAD5 |  |  | 979 | 0.213 | 0.0516 | No |
| 31 | ASPM |  |  | 980 | 0.213 | 0.0540 | No |
| 32 | PLP1 |  |  | 1022 | 0.208 | 0.0516 | No |
| 33 | PRKDC |  |  | 1051 | 0.204 | 0.0506 | No |
| 34 | BAMBI |  |  | 1144 | 0.192 | 0.0421 | No |
| 35 | EIF4A2 |  |  | 1153 | 0.191 | 0.0433 | No |
| 36 | PCSK1N |  |  | 1162 | 0.190 | 0.0445 | No |
| 37 | NASP |  |  | 1225 | 0.182 | 0.0394 | No |
| 38 | IFITM3 |  |  | 1332 | 0.168 | 0.0290 | No |
| 39 | CENPF |  |  | 1531 | 0.149 | 0.0078 | No |
| 40 | PLD3 |  |  | 1562 | 0.145 | 0.0059 | No |
| 41 | MIS18BP1 |  |  | 1765 | 0.129 | -0.0160 | No |
| 42 | BRCA2 |  |  | 1781 | 0.128 | -0.0163 | No |
| 43 | RNF19A |  |  | 1887 | 0.120 | -0.0271 | No |
| 44 | CISD2 |  |  | 1902 | 0.118 | -0.0274 | No |
| 45 | KIF20B |  |  | 1962 | 0.114 | -0.0329 | No |
| 46 | IGFBP3 |  |  | 2037 | 0.109 | -0.0403 | No |
| 47 | CEP78 |  |  | 2121 | 0.104 | -0.0487 | No |
| 48 | MXD3 |  |  | 2178 | 0.100 | -0.0540 | No |
| 49 | TRA2B |  |  | 2285 | 0.093 | -0.0653 | No |
| 50 | DIAPH3 |  |  | 2387 | 0.086 | -0.0760 | No |
| 51 | MELK |  |  | 2402 | 0.085 | -0.0766 | No |
| 52 | HAT1 |  |  | 2459 | 0.082 | -0.0822 | No |
| 53 | AKAP12 |  |  | 2478 | 0.081 | -0.0834 | No |
| 54 | ATAD2 |  |  | 2545 | 0.077 | -0.0901 | No |
| 55 | RNASEH2A |  |  | 2560 | 0.076 | -0.0909 | No |
| 56 | TMEM237 |  |  | 2680 | 0.069 | -0.1039 | No |
| 57 | ARL6IP6 |  |  | 2850 | 0.060 | -0.1227 | No |
| 58 | AURKB |  |  | 2853 | 0.060 | -0.1223 | No |
| 59 | RTKN2 |  |  | 2869 | 0.059 | -0.1234 | No |
| 60 | SYNE2 |  |  | 2881 | 0.058 | -0.1240 | No |
| 61 | RAD51AP1 |  |  | 2905 | 0.057 | -0.1260 | No |
| 62 | SRSF2 |  |  | 2908 | 0.057 | -0.1256 | No |
| 63 | CKAP2L |  |  | 2979 | 0.053 | -0.1331 | No |
| 64 | SRSF7 |  |  | 3126 | 0.046 | -0.1494 | No |
| 65 | TOMM40 |  |  | 3134 | 0.045 | -0.1497 | No |
| 66 | NCAPG |  |  | 3194 | 0.041 | -0.1561 | No |
| 67 | RFC4 |  |  | 3212 | 0.041 | -0.1576 | No |
| 68 | IGFBP5 |  |  | 3230 | 0.040 | -0.1591 | No |
| 69 | C19orf48 |  |  | 3373 | 0.033 | -0.1752 | No |
| 70 | SMC2 |  |  | 3374 | 0.032 | -0.1748 | No |
| 71 | PLAU |  |  | 3407 | 0.031 | -0.1782 | No |
| 72 | KLF6 |  |  | 3447 | 0.029 | -0.1823 | No |
| 73 | KIFC1 |  |  | 3667 | 0.021 | -0.2074 | No |
| 74 | ESCO2 |  |  | 3672 | 0.021 | -0.2076 | No |
| 75 | SMC1A |  |  | 3696 | 0.020 | -0.2101 | No |
| 76 | GTSE1 |  |  | 3738 | 0.018 | -0.2146 | No |
| 77 | PARP2 |  |  | 3831 | 0.014 | -0.2251 | No |
| 78 | NDC80 |  |  | 4034 | 0.006 | -0.2483 | No |
| 79 | MAGED2 |  |  | 4228 | -0.002 | -0.2706 | No |
| 80 | PRC1 |  |  | 4241 | -0.003 | -0.2719 | No |
| 81 | TACC3 |  |  | 4450 | -0.012 | -0.2958 | No |
| 82 | DONSON |  |  | 4454 | -0.012 | -0.2960 | No |
| 83 | HJURP |  |  | 4497 | -0.014 | -0.3007 | No |
| 84 | CDC45 |  |  | 4563 | -0.017 | -0.3081 | No |
| 85 | NUF2 |  |  | 4628 | -0.019 | -0.3152 | No |
| 86 | TCF19 |  |  | 4635 | -0.020 | -0.3157 | No |
| 87 | RNPS1 |  |  | 4641 | -0.020 | -0.3161 | No |
| 88 | CENPK |  |  | 4675 | -0.021 | -0.3196 | No |
| 89 | CDK1 |  |  | 4766 | -0.025 | -0.3298 | No |
| 90 | CENPE |  |  | 4787 | -0.025 | -0.3318 | No |
| 91 | SHCBP1 |  |  | 4848 | -0.027 | -0.3384 | No |
| 92 | NUDT15 |  |  | 4932 | -0.030 | -0.3477 | No |
| 93 | MCM7 |  |  | 4986 | -0.032 | -0.3534 | No |
| 94 | GINS2 |  |  | 5024 | -0.034 | -0.3573 | No |
| 95 | BTG1 |  |  | 5087 | -0.037 | -0.3641 | No |
| 96 | PSMC3IP |  |  | 5222 | -0.041 | -0.3791 | No |
| 97 | SPC25 |  |  | 5312 | -0.045 | -0.3889 | No |
| 98 | NUSAP1 |  |  | 5389 | -0.048 | -0.3971 | No |
| 99 | HIRIP3 |  |  | 5440 | -0.050 | -0.4024 | No |
| 100 | SPDL1 |  |  | 5460 | -0.051 | -0.4040 | No |
| 101 | ACYP1 |  |  | 5479 | -0.052 | -0.4055 | No |
| 102 | SMC3 |  |  | 5536 | -0.054 | -0.4114 | No |
| 103 | HIST2H2AC |  |  | 5542 | -0.055 | -0.4113 | No |
| 104 | TRIP13 |  |  | 5570 | -0.056 | -0.4138 | No |
| 105 | BUB3 |  |  | 5596 | -0.057 | -0.4161 | No |
| 106 | CLSPN |  |  | 5614 | -0.058 | -0.4174 | No |
| 107 | CCNA2 |  |  | 5671 | -0.061 | -0.4232 | No |
| 108 | TOP2A |  |  | 5722 | -0.063 | -0.4282 | No |
| 109 | CDCA3 |  |  | 5726 | -0.063 | -0.4279 | No |
| 110 | KIF22 |  |  | 5758 | -0.064 | -0.4308 | No |
| 111 | RMI2 |  |  | 5797 | -0.065 | -0.4344 | No |
| 112 | KPNA2 |  |  | 5811 | -0.066 | -0.4352 | No |
| 113 | CDCA4 |  |  | 5928 | -0.071 | -0.4478 | No |
| 114 | DSN1 |  |  | 5931 | -0.071 | -0.4472 | No |
| 115 | YPEL5 |  |  | 5945 | -0.072 | -0.4480 | No |
| 116 | IER2 |  |  | 5952 | -0.072 | -0.4478 | No |
| 117 | RNASEH2B |  |  | 5954 | -0.072 | -0.4472 | No |
| 118 | IL11 |  |  | 5980 | -0.073 | -0.4492 | No |
| 119 | EXOSC8 |  |  | 6020 | -0.075 | -0.4529 | No |
| 120 | FOSL1 |  |  | 6131 | -0.082 | -0.4647 | No |
| 121 | LRR1 |  |  | 6149 | -0.082 | -0.4657 | No |
| 122 | FHL2 |  |  | 6169 | -0.083 | -0.4670 | No |
| 123 | RFC5 |  |  | 6186 | -0.084 | -0.4679 | No |
| 124 | PAICS |  |  | 6198 | -0.085 | -0.4683 | No |
| 125 | PGP |  |  | 6209 | -0.086 | -0.4685 | No |
| 126 | MKI67 |  |  | 6211 | -0.086 | -0.4676 | No |
| 127 | RRM1 |  |  | 6298 | -0.090 | -0.4765 | No |
| 128 | RRM2 |  |  | 6308 | -0.091 | -0.4766 | No |
| 129 | EPS8L1 |  |  | 6346 | -0.092 | -0.4798 | No |
| 130 | RRS1 |  |  | 6354 | -0.093 | -0.4796 | No |
| 131 | CENPM |  |  | 6368 | -0.093 | -0.4801 | No |
| 132 | CKAP2 |  |  | 6372 | -0.094 | -0.4794 | No |
| 133 | CENPA |  |  | 6374 | -0.094 | -0.4784 | No |
| 134 | ASF1B |  |  | 6400 | -0.095 | -0.4803 | No |
| 135 | ARHGAP11A |  |  | 6423 | -0.097 | -0.4817 | No |
| 136 | SNRNP40 |  |  | 6441 | -0.098 | -0.4826 | No |
| 137 | ZWINT |  |  | 6453 | -0.099 | -0.4828 | No |
| 138 | DNMT1 |  |  | 6455 | -0.099 | -0.4818 | No |
| 139 | SMC4 |  |  | 6517 | -0.102 | -0.4877 | No |
| 140 | KIF4A |  |  | 6529 | -0.103 | -0.4878 | No |
| 141 | DBF4 |  |  | 6572 | -0.106 | -0.4915 | No |
| 142 | NETO2 |  |  | 6583 | -0.107 | -0.4914 | No |
| 143 | MAD2L1 |  |  | 6600 | -0.108 | -0.4921 | No |
| 144 | HNRNPDL |  |  | 6625 | -0.109 | -0.4936 | No |
| 145 | DCK |  |  | 6637 | -0.110 | -0.4937 | No |
| 146 | LMNB1 |  |  | 6638 | -0.110 | -0.4924 | No |
| 147 | VRK1 |  |  | 6651 | -0.111 | -0.4926 | No |
| 148 | CEP55 |  |  | 6700 | -0.114 | -0.4969 | No |
| 149 | DEPDC1 |  |  | 6723 | -0.115 | -0.4981 | No |
| 150 | CDCA2 |  |  | 6730 | -0.116 | -0.4975 | No |
| 151 | KIF23 |  |  | 6764 | -0.118 | -0.5000 | No |
| 152 | FBXO5 |  |  | 6775 | -0.119 | -0.4999 | No |
| 153 | ZWILCH |  |  | 6812 | -0.122 | -0.5027 | No |
| 154 | CCDC34 |  |  | 6826 | -0.123 | -0.5028 | No |
| 155 | ANLN |  |  | 6829 | -0.123 | -0.5017 | No |
| 156 | PMAIP1 |  |  | 6872 | -0.128 | -0.5051 | No |
| 157 | SESN3 |  |  | 6935 | -0.132 | -0.5108 | No |
| 158 | HSP90AA1 |  |  | 6962 | -0.134 | -0.5123 | No |
| 159 | DDIT4 |  |  | 6968 | -0.135 | -0.5114 | No |
| 160 | MND1 |  |  | 6972 | -0.135 | -0.5102 | No |
| 161 | PLP2 |  |  | 6975 | -0.135 | -0.5089 | No |
| 162 | BRIX1 |  |  | 6993 | -0.136 | -0.5094 | No |
| 163 | PBK |  |  | 7010 | -0.138 | -0.5097 | No |
| 164 | DNAJC9 |  |  | 7043 | -0.141 | -0.5118 | No |
| 165 | RBBP8 |  |  | 7099 | -0.145 | -0.5166 | No |
| 166 | MIS18A |  |  | 7115 | -0.147 | -0.5167 | No |
| 167 | CENPW |  |  | 7116 | -0.147 | -0.5150 | No |
| 168 | KPNB1 |  |  | 7144 | -0.150 | -0.5165 | No |
| 169 | RPL22L1 |  |  | 7150 | -0.150 | -0.5154 | No |
| 170 | TFDP1 |  |  | 7187 | -0.154 | -0.5178 | No |
| 171 | FEN1 |  |  | 7201 | -0.155 | -0.5176 | No |
| 172 | SDCBP |  |  | 7222 | -0.158 | -0.5182 | No |
| 173 | GMNN |  |  | 7224 | -0.158 | -0.5165 | No |
| 174 | HSPB11 |  |  | 7286 | -0.166 | -0.5217 | No |
| 175 | TMPO |  |  | 7296 | -0.167 | -0.5209 | No |
| 176 | SUPT16H |  |  | 7322 | -0.170 | -0.5219 | No |
| 177 | CENPH |  |  | 7329 | -0.171 | -0.5207 | No |
| 178 | EIF2S1 |  |  | 7366 | -0.177 | -0.5229 | No |
| 179 | SNRPA1 |  |  | 7376 | -0.178 | -0.5219 | No |
| 180 | TK1 |  |  | 7377 | -0.178 | -0.5199 | No |
| 181 | TUBB6 |  |  | 7396 | -0.180 | -0.5200 | No |
| 182 | USP1 |  |  | 7398 | -0.181 | -0.5181 | No |
| 183 | CMSS1 |  |  | 7453 | -0.187 | -0.5223 | No |
| 184 | PSIP1 |  |  | 7460 | -0.187 | -0.5209 | No |
| 185 | CENPN |  |  | 7528 | -0.194 | -0.5265 | Yes |
| 186 | CMC2 |  |  | 7539 | -0.196 | -0.5254 | Yes |
| 187 | MAGOHB |  |  | 7542 | -0.196 | -0.5235 | Yes |
| 188 | CKLF |  |  | 7547 | -0.197 | -0.5217 | Yes |
| 189 | TPX2 |  |  | 7549 | -0.197 | -0.5197 | Yes |
| 190 | MCM3 |  |  | 7552 | -0.198 | -0.5177 | Yes |
| 191 | UBE2T |  |  | 7561 | -0.199 | -0.5164 | Yes |
| 192 | BEX4 |  |  | 7566 | -0.199 | -0.5146 | Yes |
| 193 | SAE1 |  |  | 7570 | -0.200 | -0.5128 | Yes |
| 194 | BIRC5 |  |  | 7596 | -0.204 | -0.5134 | Yes |
| 195 | BTG3 |  |  | 7602 | -0.204 | -0.5117 | Yes |
| 196 | DERA |  |  | 7626 | -0.207 | -0.5120 | Yes |
| 197 | TEX30 |  |  | 7639 | -0.209 | -0.5111 | Yes |
| 198 | CARHSP1 |  |  | 7667 | -0.213 | -0.5118 | Yes |
| 199 | WDR83OS |  |  | 7685 | -0.215 | -0.5114 | Yes |
| 200 | NXT1 |  |  | 7688 | -0.217 | -0.5092 | Yes |
| 201 | CDKN2D |  |  | 7721 | -0.222 | -0.5104 | Yes |
| 202 | HAUS1 |  |  | 7761 | -0.227 | -0.5124 | Yes |
| 203 | EIF5 |  |  | 7763 | -0.227 | -0.5100 | Yes |
| 204 | UBE2I |  |  | 7770 | -0.228 | -0.5082 | Yes |
| 205 | FBL |  |  | 7780 | -0.230 | -0.5067 | Yes |
| 206 | SAC3D1 |  |  | 7825 | -0.240 | -0.5091 | Yes |
| 207 | PXMP2 |  |  | 7827 | -0.240 | -0.5065 | Yes |
| 208 | MZT1 |  |  | 7838 | -0.242 | -0.5050 | Yes |
| 209 | TUBG1 |  |  | 7846 | -0.243 | -0.5031 | Yes |
| 210 | UPP1 |  |  | 7849 | -0.243 | -0.5006 | Yes |
| 211 | WDR34 |  |  | 7862 | -0.246 | -0.4992 | Yes |
| 212 | CENPU |  |  | 7871 | -0.248 | -0.4974 | Yes |
| 213 | DHFR |  |  | 7878 | -0.250 | -0.4953 | Yes |
| 214 | PSMG1 |  |  | 7880 | -0.250 | -0.4926 | Yes |
| 215 | ZFAND6 |  |  | 7909 | -0.257 | -0.4930 | Yes |
| 216 | COPS3 |  |  | 7912 | -0.258 | -0.4904 | Yes |
| 217 | SOX4 |  |  | 7958 | -0.268 | -0.4926 | Yes |
| 218 | H2AFX |  |  | 7965 | -0.269 | -0.4903 | Yes |
| 219 | CKS2 |  |  | 7972 | -0.270 | -0.4880 | Yes |
| 220 | HMGB2 |  |  | 7973 | -0.271 | -0.4850 | Yes |
| 221 | HPRT1 |  |  | 7974 | -0.271 | -0.4819 | Yes |
| 222 | HIST1H1C |  |  | 7978 | -0.271 | -0.4793 | Yes |
| 223 | CENPV |  |  | 8000 | -0.276 | -0.4786 | Yes |
| 224 | MYBL2 |  |  | 8041 | -0.283 | -0.4801 | Yes |
| 225 | RFC2 |  |  | 8049 | -0.285 | -0.4777 | Yes |
| 226 | CHCHD3 |  |  | 8055 | -0.287 | -0.4751 | Yes |
| 227 | MAP3K7CL |  |  | 8063 | -0.288 | -0.4727 | Yes |
| 228 | DTYMK |  |  | 8075 | -0.292 | -0.4707 | Yes |
| 229 | CTNNAL1 |  |  | 8076 | -0.292 | -0.4675 | Yes |
| 230 | PCNA |  |  | 8082 | -0.293 | -0.4648 | Yes |
| 231 | RBM17 |  |  | 8110 | -0.299 | -0.4646 | Yes |
| 232 | DUT |  |  | 8118 | -0.300 | -0.4620 | Yes |
| 233 | RPS27L |  |  | 8126 | -0.302 | -0.4595 | Yes |
| 234 | POLR3K |  |  | 8132 | -0.305 | -0.4566 | Yes |
| 235 | TMEM106C |  |  | 8146 | -0.308 | -0.4547 | Yes |
| 236 | SAT1 |  |  | 8162 | -0.314 | -0.4530 | Yes |
| 237 | HIST1H1E |  |  | 8176 | -0.320 | -0.4509 | Yes |
| 238 | SSRP1 |  |  | 8178 | -0.320 | -0.4475 | Yes |
| 239 | PARP1 |  |  | 8182 | -0.321 | -0.4442 | Yes |
| 240 | SRSF3 |  |  | 8191 | -0.323 | -0.4416 | Yes |
| 241 | HMGN1 |  |  | 8192 | -0.324 | -0.4379 | Yes |
| 242 | CKS1B |  |  | 8241 | -0.336 | -0.4397 | Yes |
| 243 | PSMC3 |  |  | 8261 | -0.342 | -0.4381 | Yes |
| 244 | ENC1 |  |  | 8274 | -0.346 | -0.4357 | Yes |
| 245 | UBE2C |  |  | 8278 | -0.348 | -0.4321 | Yes |
| 246 | DCTPP1 |  |  | 8303 | -0.356 | -0.4309 | Yes |
| 247 | EIF4EBP1 |  |  | 8306 | -0.356 | -0.4272 | Yes |
| 248 | HNRNPF |  |  | 8319 | -0.360 | -0.4246 | Yes |
| 249 | PA2G4 |  |  | 8320 | -0.360 | -0.4206 | Yes |
| 250 | SOX9 |  |  | 8327 | -0.361 | -0.4173 | Yes |
| 251 | EIF1AX |  |  | 8331 | -0.363 | -0.4136 | Yes |
| 252 | HSPA8 |  |  | 8339 | -0.366 | -0.4103 | Yes |
| 253 | TUBB4B |  |  | 8356 | -0.371 | -0.4080 | Yes |
| 254 | MRPL18 |  |  | 8358 | -0.373 | -0.4040 | Yes |
| 255 | NUDT1 |  |  | 8359 | -0.373 | -0.3998 | Yes |
| 256 | HOXB2 |  |  | 8363 | -0.375 | -0.3960 | Yes |
| 257 | HSPD1 |  |  | 8418 | -0.393 | -0.3978 | Yes |
| 258 | SNRPE |  |  | 8435 | -0.401 | -0.3952 | Yes |
| 259 | HNRNPD |  |  | 8436 | -0.402 | -0.3907 | Yes |
| 260 | HIST1H1B |  |  | 8437 | -0.402 | -0.3863 | Yes |
| 261 | HNRNPAB |  |  | 8441 | -0.404 | -0.3821 | Yes |
| 262 | PTTG1 |  |  | 8446 | -0.406 | -0.3780 | Yes |
| 263 | PFDN2 |  |  | 8465 | -0.417 | -0.3755 | Yes |
| 264 | LYPD1 |  |  | 8476 | -0.422 | -0.3719 | Yes |
| 265 | CACYBP |  |  | 8486 | -0.428 | -0.3682 | Yes |
| 266 | GGCT |  |  | 8493 | -0.432 | -0.3641 | Yes |
| 267 | SNRNP25 |  |  | 8494 | -0.432 | -0.3593 | Yes |
| 268 | NCL |  |  | 8496 | -0.433 | -0.3546 | Yes |
| 269 | TMEM158 |  |  | 8499 | -0.434 | -0.3500 | Yes |
| 270 | ST13 |  |  | 8512 | -0.442 | -0.3464 | Yes |
| 271 | HIST1H1D |  |  | 8520 | -0.443 | -0.3423 | Yes |
| 272 | RAD23A |  |  | 8524 | -0.446 | -0.3377 | Yes |
| 273 | MRPL17 |  |  | 8528 | -0.446 | -0.3331 | Yes |
| 274 | SNRPD1 |  |  | 8537 | -0.451 | -0.3290 | Yes |
| 275 | C12orf75 |  |  | 8546 | -0.456 | -0.3248 | Yes |
| 276 | RAN |  |  | 8555 | -0.463 | -0.3206 | Yes |
| 277 | RPA3 |  |  | 8572 | -0.471 | -0.3172 | Yes |
| 278 | SNRPG |  |  | 8578 | -0.473 | -0.3125 | Yes |
| 279 | PRDX1 |  |  | 8584 | -0.476 | -0.3078 | Yes |
| 280 | PTGES3 |  |  | 8587 | -0.479 | -0.3027 | Yes |
| 281 | RAB13 |  |  | 8590 | -0.481 | -0.2975 | Yes |
| 282 | TYMS |  |  | 8599 | -0.488 | -0.2930 | Yes |
| 283 | POP7 |  |  | 8605 | -0.495 | -0.2881 | Yes |
| 284 | HMGN2 |  |  | 8608 | -0.496 | -0.2828 | Yes |
| 285 | PIN1 |  |  | 8623 | -0.509 | -0.2788 | Yes |
| 286 | CYCS |  |  | 8640 | -0.519 | -0.2748 | Yes |
| 287 | HSPE1 |  |  | 8650 | -0.524 | -0.2700 | Yes |
| 288 | HMGB1 |  |  | 8653 | -0.527 | -0.2644 | Yes |
| 289 | SIVA1 |  |  | 8654 | -0.529 | -0.2585 | Yes |
| 290 | EEF2 |  |  | 8670 | -0.540 | -0.2542 | Yes |
| 291 | HMGA1 |  |  | 8675 | -0.543 | -0.2486 | Yes |
| 292 | ID3 |  |  | 8676 | -0.544 | -0.2426 | Yes |
| 293 | CDKN1A |  |  | 8679 | -0.545 | -0.2367 | Yes |
| 294 | ANP32B |  |  | 8680 | -0.545 | -0.2307 | Yes |
| 295 | SLC25A5 |  |  | 8681 | -0.546 | -0.2246 | Yes |
| 296 | SNRPB |  |  | 8683 | -0.547 | -0.2186 | Yes |
| 297 | H2AFV |  |  | 8693 | -0.554 | -0.2135 | Yes |
| 298 | RHEB |  |  | 8694 | -0.554 | -0.2073 | Yes |
| 299 | MLLT11 |  |  | 8695 | -0.555 | -0.2011 | Yes |
| 300 | C4orf3 |  |  | 8705 | -0.565 | -0.1959 | Yes |
| 301 | YWHAH |  |  | 8726 | -0.587 | -0.1916 | Yes |
| 302 | PDCD5 |  |  | 8729 | -0.592 | -0.1853 | Yes |
| 303 | PFDN5 |  |  | 8738 | -0.596 | -0.1796 | Yes |
| 304 | CALM2 |  |  | 8744 | -0.600 | -0.1735 | Yes |
| 305 | TUBB |  |  | 8763 | -0.621 | -0.1686 | Yes |
| 306 | ATP6V1G1 |  |  | 8766 | -0.625 | -0.1619 | Yes |
| 307 | DEK |  |  | 8770 | -0.627 | -0.1552 | Yes |
| 308 | COMMD6 |  |  | 8772 | -0.628 | -0.1484 | Yes |
| 309 | H2AFZ |  |  | 8774 | -0.630 | -0.1415 | Yes |
| 310 | STMN1 |  |  | 8783 | -0.638 | -0.1353 | Yes |
| 311 | PHLDA1 |  |  | 8805 | -0.655 | -0.1304 | Yes |
| 312 | FABP7 |  |  | 8811 | -0.661 | -0.1236 | Yes |
| 313 | SET |  |  | 8813 | -0.664 | -0.1163 | Yes |
| 314 | RANBP1 |  |  | 8819 | -0.669 | -0.1095 | Yes |
| 315 | BEX1 |  |  | 8833 | -0.689 | -0.1033 | Yes |
| 316 | SMS |  |  | 8834 | -0.690 | -0.0956 | Yes |
| 317 | PRELID1 |  |  | 8847 | -0.705 | -0.0891 | Yes |
| 318 | HIST1H4C |  |  | 8850 | -0.710 | -0.0815 | Yes |
| 319 | LSM4 |  |  | 8853 | -0.712 | -0.0738 | Yes |
| 320 | RPL34 |  |  | 8877 | -0.744 | -0.0681 | Yes |
| 321 | TUBA1A |  |  | 8883 | -0.751 | -0.0604 | Yes |
| 322 | PTMA |  |  | 8894 | -0.764 | -0.0530 | Yes |
| 323 | CHCHD2 |  |  | 8898 | -0.770 | -0.0448 | Yes |
| 324 | UBE2S |  |  | 8901 | -0.772 | -0.0364 | Yes |
| 325 | S100B |  |  | 8908 | -0.784 | -0.0284 | Yes |
| 326 | EEF1A1 |  |  | 8958 | -0.915 | -0.0238 | Yes |
| 327 | TUBA1B |  |  | 8980 | -1.103 | -0.0140 | Yes |
| 328 | FTL |  |  | 8984 | -1.286 | 0.0000 | Yes |
Table: GSEA details [plain text format]

  

Fig 2: FELDMAN\_2020\_S-G2: Random ES distribution      
 Gene set null distribution of ES for **FELDMAN\_2020\_S-G2**
